# Fibroblast growth factor 23 in acute myocardial infarction complicated by cardiogenic shock: a biomarker substudy of the Intraaortic Balloon Pump in Cardiogenic Shock II (IABP-SHOCK II) trial

**DOI:** 10.1186/s13054-014-0713-8

**Published:** 2014-12-21

**Authors:** Georg Fuernau, Janine Pöss, Daniel Denks, Steffen Desch, Gunnar H Heine, Ingo Eitel, Sarah Seiler, Suzanne de Waha, Sebastian Ewen, Andreas Link, Gerhard Schuler, Volker Adams, Michael Böhm, Holger Thiele

**Affiliations:** Clinic for Internal Medicine/Cardiology, University of Leipzig - Heart Center, Strümpellstraße 39, 04289 Leipzig, Germany; Medical Clinic II, Universitätsklinikum Schleswig-Holstein (UKSH), Campus Lübeck, Ratzeburger Allee 160, 23538 Lübeck, Germany; Department of Internal Medicine III - Cardiology, Angiology, and Intensive Care Medicine, Saarland University Medical Center and Saarland University Faculty of Medicine, Kirrberger Straße 100, 66421 Homburg, Germany; Internal Medicine IV - Nephrology and Hypertension, Clinic for Internal Medicine IV - Nephrology and Hypertension, Saarland University Medical Center and Saarland University Faculty of Medicine, Kirrberger Straße 100, 66421 Homburg, Germany; Department of Cardiology, Heart Center Segeberger Kliniken GmbH, Am Kurpark 1, 23795 Bad Segeberg, Germany

## Abstract

**Introduction:**

Cardiogenic shock (CS) is the leading cause of death in patients hospitalized with acute myocardial infarction (AMI). Biomarkers might help in risk stratification and understanding of pathophysiology. Preliminary data suggests that patients with CS face a profound increase in the osteocyte-derived hormone fibroblast growth factor 23 (FGF-23), which acts as a negative regulator of serum phosphate levels. The present study aimed to assess the predictive role of FGF-23 for clinical outcome in a large cohort of CS patients with and without renal dysfunction.

**Methods:**

In the randomized Intraaortic Balloon Pump in Cardiogenic Shock II (IABP-SHOCK II) trial, 600 patients with CS complicating AMI were assigned to therapy with or without IABP. Our predefined biomarker substudy included 182 patients. Blood sampling was performed in a standardized procedure at three different time points (day 1 (day of admission), day 2 and day 3). Differences in outcome of patients with FGF-23 levels < and > median were compared by log-rank testing. Stepwise logistic regression modeling was performed to identify predictors of death at 30 days and Cox regression analysis for time to death during the first year.

**Results:**

At all three time points, nonsurvivors had significantly higher FGF-23 levels compared to survivors (*P* <0.001 for all). Patients with FGF-23 levels above the median (395 RU/mL [interquartile range 102;2,395]) were characterized by an increased 30-day mortality and 1-year mortality. In multivariable analysis FGF-23 levels remained independent predictors for 30-day (odds ratio per 10log 1.80, 95% confidence interval (CI) 1.11 to 2.92; *P* = 0.02) and 1-year mortality (hazard ratio 1.50, 95% CI 1.11 to 2.04, *P* = 0.009). After stratifying the patients according to their baseline serum creatinine levels, the negative prognostic association of increased FGF-23 was only significant in those with serum creatinine greater than median.

**Conclusions:**

In CS, high levels of FGF-23 are independently related to a poor clinical outcome. However, this prognostic association appears only to apply in patients with impaired renal function.

**Trial registration:**

ClinicalTrials.gov NCT00491036. Registered 22 June 2007.

## Introduction

Cardiogenic shock (CS) represents the leading cause of death in patients hospitalized with acute myocardial infarction (AMI) [1-3]. Despite advances in treatment over the last decades, mortality rates are still approaching 50%. The pathophysiology of CS is characterized by an activation of neurohormones and inflammatory markers, which contribute to a vicious spiral resulting in systemic inflammatory response syndrome and finally leading to death [4].

Epidemiologic studies indicate that hyperphosphatemia is related to cardiovascular morbidity and mortality. This relationship has first been observed in patients with chronic kidney disease (CKD), but interestingly, it was found also in the general population [5]. Fibroblast growth factor 23 (FGF-23) is a phosphaturic hormone produced by osteocytes [6]. It acts by reducing phosphate absorption in the intestinal tract, increasing renal phosphate excretion, and lowering renal vitamin D activation [7]. Furthermore, FGF-23 seems to be upregulated due to activation of the renin-angiotensin-aldosterone-system (RAAS) [8], which may explain the high levels of FGF-23 observed in CS [9]. Serum levels of FGF-23 have been shown to predict cardiovascular events in patients with CKD [10,11], in patients with normal renal function and a prevalent cardiovascular disease [12] and also among individuals from the general population [13,14]. Very recent evidence suggests that, within the broad spectrum of cardiovascular diseases, FGF-23 might be a much stronger predictor of incident heart failure events rather than of atherosclerotic vascular events, both among CKD patients, [10,11] and among subjects with a preserved renal function [15].

This close association between FGF-23 and myocardial disease was further confirmed in two recent cohort studies that selectively recruited patients with stable chronic systolic heart failure, among whom FGF-23 independently predicted cardiovascular events and total mortality [12,13].

In line, several clinical studies associated FGF-23 with markers of myocardial damage, namely left ventricular hypertrophy [16-18] and left ventricular dysfunction [19]. However, it is still controversial whether these associations are causal and whether FGF-23 exerts direct effects on the cardiovascular system [16,20].

A prior small study found that in patients with AMI complicated by CS without apparent pre-existing CKD FGF-23 levels were profoundly increased, showing more than 10-fold higher FGF-23 levels in patients with CS compared to patients with stable coronary artery disease (CAD). Furthermore, increased FGF-23 concentrations were associated with worse clinical outcome [9]. Notably, FGF-23 levels in patients with uncomplicated AMI did not differ from those of stable CAD patients [9]. This observed increase in CS patients exceeded by far the one observed in patients with hyperphosphatemia due to CKD and thus, seemed to occur largely (if not completely) independently of the serum phosphate levels of the patients and suggests that - at least under certain circumstances - CS may induce elevated FGF-23 levels. Limitations of this preliminary study were its small sample size with low numbers of events and that the prognostic role of FGF-23 was not assessed with regard to renal function.

The present study aimed to further assess the prognostic association of FGF-23 in a larger, better characterized population with infarction-related CS included in a prospective, randomized clinical trial and to investigate its relationship to renal function.

## Methods

### Patients and study design

**Figure 1:**
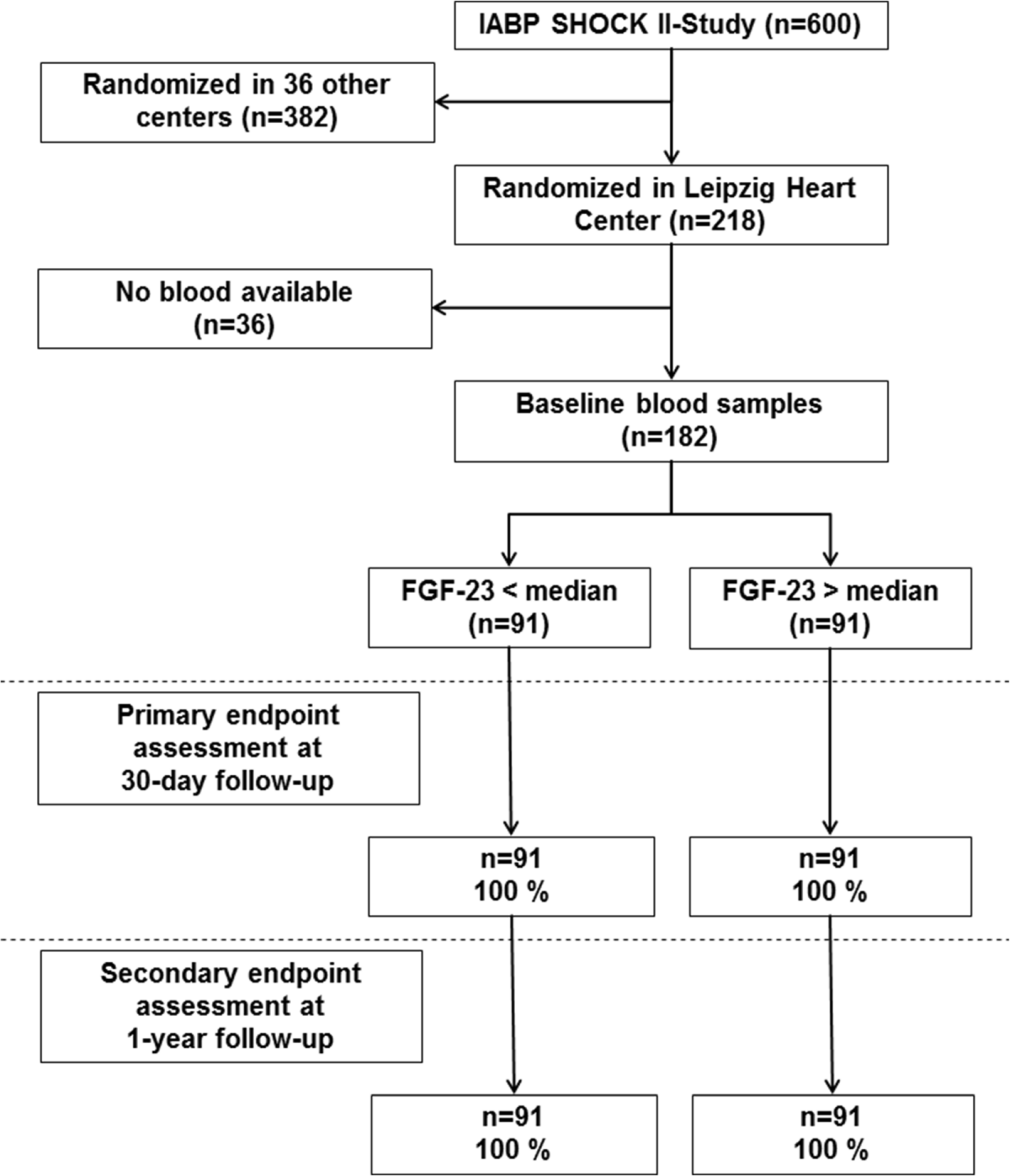
**Study flow.** FGF-23, fibroblast growth factor 23.

The present study is a predefined substudy of the Intraaortic Balloon Pump in Cardiogenic Shock II trial (IABP-SHOCK II; ClinicalTrials.gov Identifier: NCT00491036) comparing the use of intraaortic balloon pump (IABP) versus no IABP support in patients with CS complicating AMI. This study did not show a significant difference between the two groups with respect to short- and long-term outcome. The design of the trial and its main results have been published previously [21-23]. In brief, 600 patients were enrolled in 37 centers in Germany and were randomized to either IABP support or to control in a 1:1 fashion. CS was defined as hypotension, pulmonary congestion, and signs of end-organ hypoperfusion. Exclusion criteria were duration of CS >12 hours, cardiopulmonary resuscitation >30 minutes, severe cerebral deficit, mechanical causes of CS, age >90 years, absolute contraindications against IABP insertion, shock of other cause, or severe concomitant disease with life expectancy <6 months. Of the 600 patients included in the trial, 218 were enrolled at the University of Leipzig - Heart Center with a prospectively planned serial blood sampling. From 182 of these patients, blood samples were available for the present analysis (Figure 1). All patients underwent cardiac catheterization immediately after hospital admission. In resuscitated patients, cooling was initiated after percutaneous coronary intervention (PCI) and blood sampling. The study was conducted according to the Declaration of Helsinki, has been approved by the ethical board of the medical faculty at the University of Leipzig and all patients or their legal representatives gave written informed consent.

### Laboratory measurements

Blood samples were collected under standardized conditions at different, predefined time points (day 1 (day of admission during primary PCI), day 2 and day 3). Samples were immediately centrifuged at 2,400 g for 10 minutes at room temperature. Supernatants were stored in aliquots at −80°C until further use. C-terminal FGF-23 was measured from EDTA-plasma samples by enzyme-linked immunosorbent assay (FGF-23: Immunotopics, San Clemente, CA, USA; low cutoff value 3 rU/mL, high cutoff value 2,000 rU/mL. Samples with FGF-23 > 2,000 rU/mL were measured after dilution). Samples were assayed in duplicate. Plasma levels of creatinine, serum lactate and troponin T were measured by standard institutional laboratory measurements. For assessment of the association of FGF-23 levels with outcome with respect to renal function, the patient cohort was divided by the median of baseline serum creatinine.

### Statistical analysis

Categorical data are presented as counts or proportions with the corresponding percentages and compared by chi^2^ test. Most continuous variables were not normally distributed. For reasons of uniformity, summary statistics for all continuous variables are thus presented as medians with interquartile range (IQR). For comparison of continuous variables, Student’s *t* test or Mann–Whitney test were used, as appropriate. Spearman’s rank correlation was used to investigate associations between continuous variables. For outcome analysis, all-cause mortality at 30 days and 1 year was used. Patients were stratified into two groups according to the median of FGF-23 concentrations at baseline. Log-rank testing was used for outcome assessment. Stepwise logistic regression modeling for short-term (30-day) mortality and Cox-regression modeling for long-term (1-year) mortality were performed. All baseline variables with an association (*P* value <0.1) to short- or long-term mortality in univariable analysis entered the specific model. To assess the association between kidney function and increased FGF-23 levels at baseline (> vs. < median), patients were stratified in two groups according to the median of serum creatinine concentrations at baseline and the odds ratios (ORs) were assessed separately including the calculation for interaction. Finally, the diagnostic accuracy of FGF-23 for the prediction of mortality was explored by receiver-operating-characteristics curve analysis. Comparison of the area under the curves (AUC) of FGF-23, serum lactate and a combination of both factors by multivariable logistic regression was performed with c statistics. Statistical analysis was performed using commercially available software (MedCalc for Windows, version 13.3.1.0; MedCalc Software, Ostend, Belgium). A two-tailed *P* value <0.05 was considered statistically significant.

## Results

The baseline characteristics of patients with FGF-23 concentrations at baseline below and above the median (median: 395 RU/mL, IQR 102;2,395) are depicted in Table 1. As compared to patients < median, those with FGF-23 > median were older, had worse renal function, higher serum lactate and troponin T concentrations. In addition, they were more likely to be female, to have prevalent peripheral artery disease, and/or coronary triple-vessel disease. Of the 182 CS patients, 73 (40.1%) died within 30 days and 97 (53.3%) within 1 year.

**Table 1:** **Baseline characteristics**

	**Overall**	**FGF-23 < median**	**FGF-23 > median**	***P*** **value**
	**n = 182**	**n = 91**	**n = 91**	
Age (years)	71 (58;79)	67 (54;75)	74 (63;81)	<0.001
Male sex, n (%)	126 (69)	71 (78)	55 (60)	0.02
Body mass index (kg/m^2^)	27.3 (24.5;29.4)	27.5 (24.7;29.7)	27.1 (24.5;29.4)	0.49
Baseline serum creatinine (μmol/L)	117 (95;163)	99 (81;123)	147 (109;208)	<0.001
Baseline serum lactate (mmol/L)	3.7 (2.3;7.1)	3.0 (2.0;5.5)	4.6 (3.1;7.8)	<0.001
Baseline troponin T (μg/L)	0.9 (0.3;2.9)	0.7 (0.2;1.6)	1.2 (0.4;3.4)	0.01
Heart rate at admission (n/min)	91 (75;110)	90 (72;110)	98 (78;114)	0.16
Systolic blood pressure at admission (mmHg)	86 (78;106)	85 (79;105)	86 (78;106)	0.98
Hypertension, n (%)	127 (70)	57 (63)	70 (77)	0.053
Hypercholesterolemia, n (%)	55 (30)	22 (24)	33 (36)	0.11
Diabetes mellitus, n (%)	65 (36)	28 (31)	37 (41)	0.22
Known peripheral artery disease, n (%)	22 (12)	5 (5)	17 (19)	0.01
Prior myocardial infarction, n (%)	39 (21)	20 (22)	19 (21)	0.99
Prior PCI, n (%)	33 (18)	17 (19)	16 (18)	0.99
Prior CABG, n (%)	10 (6)	6 (7)	4 (4)	0.75
Randomized to IABP, n (%)	89 (49)	51 (56)	38 (42)	0.08
Triple-vessel disease, n (%)	90 (50)	33 (36)	57 (63)	<0.001
Resuscitation, n (%)	67 (37)	38 (42)	29 (32)	0.22
Mechanical ventilation at admission, n (%)	95 (52)	45 (49)	50 (55)	0.55

FGF-23: fibroblast growth factor-23; PCI: percutaneous coronary intervention; CABG: coronary artery bypass graft; IABP: intraaortic balloon pump.

### Association of FGF-23 with clinical outcome

**Figure 2:**
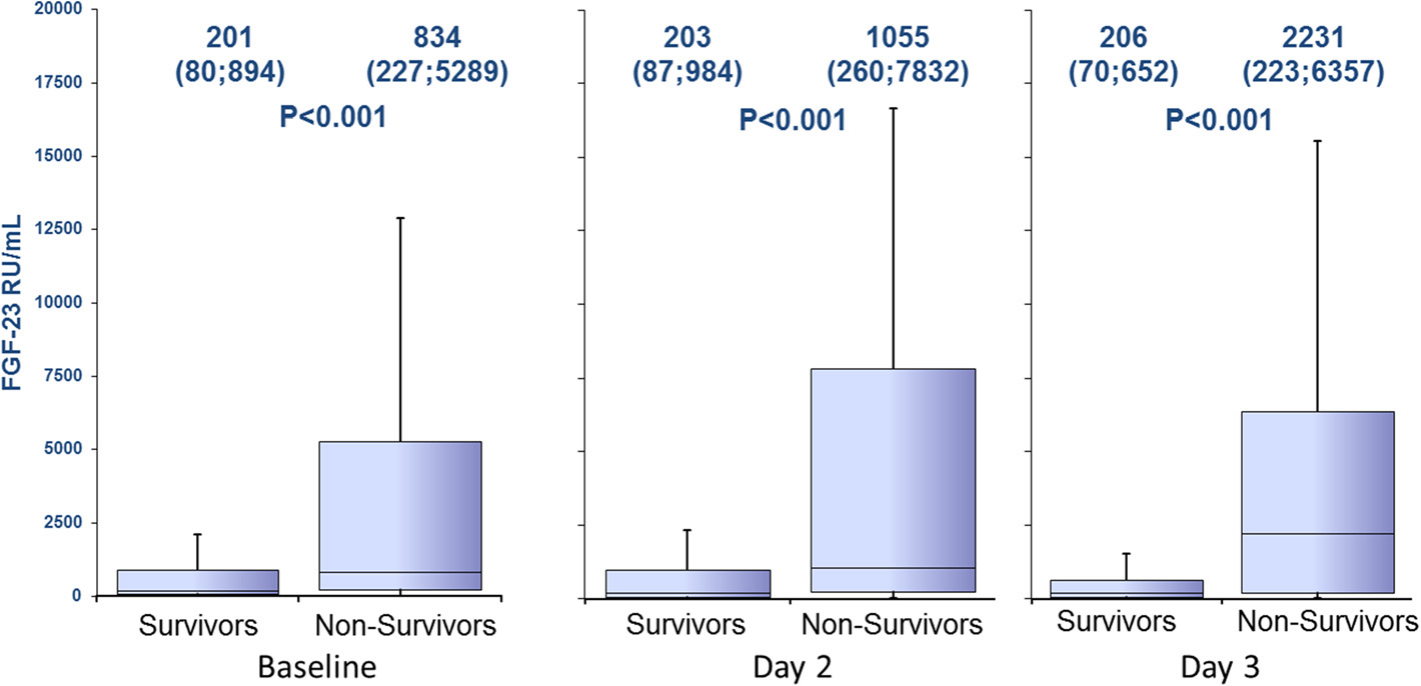
**Levels of FGF-23 on the different time points for 30-day survivors and nonsurvivors.** FGF-23: fibroblast growth factor 23.

**Figure 3:**
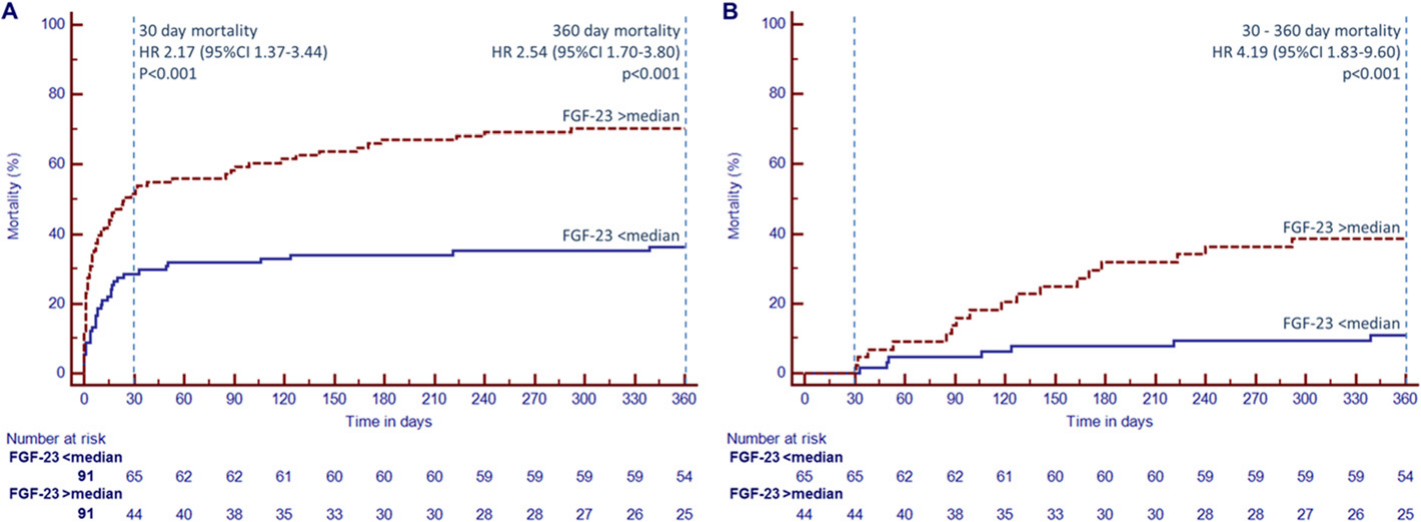
**Kaplan-Meier curves for patients with FGF-23 levels > median (red dashed line) and < median (blue line) for the overall cohort (A) and as landmark analysis for patients surviving until day 30 (B).** FGF-23: fibroblast growth factor 23; HR: hazard ratio; CI: confidence interval.

Nonsurvivors after 30 days had significantly higher FGF-23 levels compared to survivors; this difference was even more accentuated at day 2 and day 3 (Figure 2). In Kaplan-Meier analysis, patients with baseline FGF-23 levels above the median were characterized by an increased 30-day and 1-year mortality (Figure 3A). A landmark analysis in the 30-day survivors showed that a mortality difference was also found after the acute phase (Figure 3B). Also FGF-23 levels on day 2 and 3 were predictive in Kaplan-Meier analysis for short-term (day 2: hazard ratio (HR) 2.41, 95% confidence interval (CI) 1.29 to 4.49, *P* = 0.007, day 3: HR 2.89, 95%CI 1.56 to 5.34, *P* = 0.001), and long-term mortality (day 2: HR 2.31, 95%CI 1.41 to 3.80, *P* = 0.001, day 3: HR 3.13, 95%CI 1.91 to 5.14, *P* <0.001).

### Regression analysis

In univariable logistic regression analysis FGF-23, age, ejection fraction (EF), serum lactate and serum creatinine concentrations showed an association with time to 30-day mortality (*P* <0.1 for all). In multivariable stepwise logistic regression analysis including these parameters, FGF-23 concentration remained an independent predictor of clinical outcome at 30 days (Table 2). In long-term (1-year) survival analysis including the same parameters, FGF-23 levels also remained independent predictors for time to death (independent predictors: FGF-23 per 10LOG: HR 1.50, 95%CI 1.11 to 2.04, *P* = 0.009, age per 10 years: HR 1.27, 95%CI 1.03 to 1.56, *P* = 0.03; EF per 10% increase: HR 0.76, 95%CI 0.62 to 0.92, *P* = 0.006; serum lactate per 10LOG: HR 2.92, 95%CI 1.23 to 6.94, *P* = 0.009, coronary three-vessel disease and baseline serum creatinine, both univariable predictive, were not significant in multivariable testing).

**Table 2:** **Multivariable stepwise logistic regression analysis for 30-day mortality**

	**Univariable analysis**			**Multivariable stepwise logistic regression analysis**		
	**OR**	**95%CI**	***P***	**OR**	**95%CI**	***P***
FGF-23 per 10LOG	2.08	1.41–3.06	<0.001	1.80	1.11–2.92	0.02
Age per 10 years	1.30	1.02–1.66	0.03	-	-	-
Ejection fraction per 10%	0.59	0.44–0.80	<0.001	0.63	0.46–0.86	0.003
Diabetes mellitus	1.21	0.65–2.24	0.54			
Serum lactate per 10LOG	7.90	2.79–22.42	<0.001	4.18	1.17–14.87	0.03
Serum creatinine per 10LOG	15.35	2.92–80.77	0.001	-	-	-
Troponin T per 10LOG	1.29	0.80–2.07	0.30			
Known peripheral artery disease	1.28	0.52–3.15	0.59			
Systolic blood pressure per 10 mmHg	0.93	0.81–1.08	0.33			
Randomized to control group	1.13	0.62–2.04	0.69			
Female gender	1.46	0.77–2.76	0.25			
Three-vessel disease	1.57	0.86–2.85	0.14			
Resuscitation prior randomization	1.36	0.74–2.50	0.33			
Mechanical ventilation at admission.	1.43	0.79–2.60	0.24			

OR: odds ratio; CI: confidence interval; FGF-23: fibroblast growth factor 23.

### Receiver operating characteristics

In c statistics, the AUC of FGF-23 levels was equal to the one of serum lactate concentrations at baseline with regard to the prediction of 30-day mortality (FGF-23 vs. serum lactate: 0.677 vs. 0.673; *P* = 0.95). Interestingly, when combining both factors, the AUC increased significantly compared to the AUC of serum lactate alone (0.724 vs. 0.673; *P* = 0.04).

### FGF-23 and renal function

**Figure 4:**
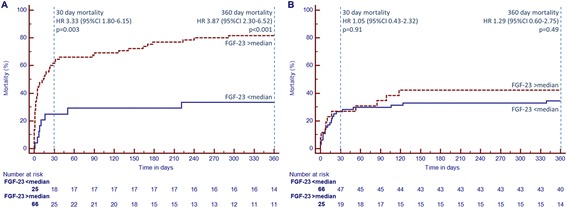
**Kaplan-Meier curves for patients with FGF-23 levels > median (red dashed line) and < median (blue line) for patients with baseline serum creatinine > median (A) and < median (B).** FGF-23: fibroblast growth factor 23; HR: hazard ratio; CI: confidence interval.

**Figure 5:**
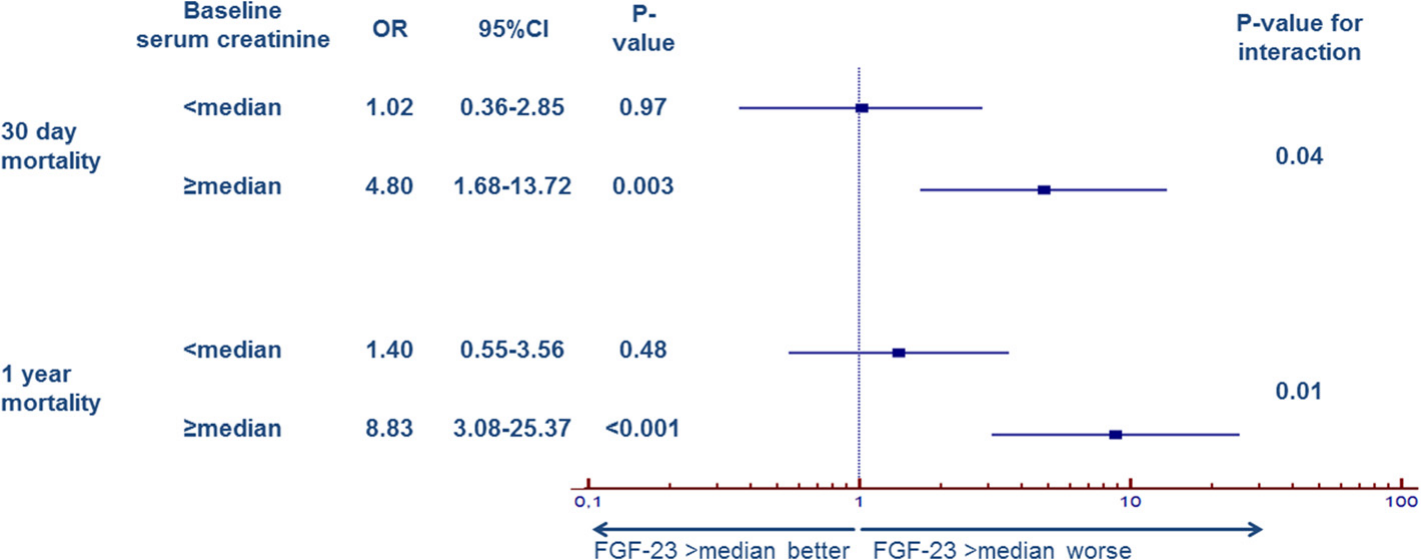
**Forest plot for odds ratios of FGF-23 levels > median for short- and long-term survival grouped by baseline serum creatinine > median and < median.** FGF-23: fibroblast growth factor 23; OR: odds ratio; CI: confidence interval.

Baseline FGF-23 levels correlated moderately with baseline serum creatinine levels (r = 0.583; *P* <0.001). Patients were stratified in two groups according to the median creatinine levels at baseline (117 μmol/L). FGF-23 levels at baseline were significantly higher in patients with serum creatinine levels > median (low vs. high creatinine: 150 [IQR 69; 485] vs. 1264 [358; 5706] RU/mL, *P* <0.001). The difference in FGF-23 concentrations between survivors and nonsurvivors was only evident in the group of patients with creatinine concentrations above the median (FGF-23: 626 [IQR 164;3,673] vs. 3,477 [690;9,986] RU/mL, *P* <0.001), whereas in the group with creatinine concentrations below the median, no differences in FGF-23 levels were observed with respect to the survival of the patients (141 (72;458) vs. 204 (62;524) RU/mL, *P* = 0.72). In accordance, the negative prognostic association of increased baseline FGF-23 with 30-day mortality as well as 1-year mortality was only significant in patients with serum creatinine above the median, whereas in the group of patients with creatinine below the median, no difference was observed (Figure 4A + B). This was also observed for the adjusted HR for long-term mortality by quartiles of FGF-23 concentration (Table 3). The calculated interaction terms for baseline FGF-23 < and > median with baseline serum creatinine < and > median in prediction of short- (*P* = 0.04) and long-term (*P* = 0.01) survival were significant (Figure 5). When the population was divided into tertiles by baseline serum creatinine the AUC for the prognostic value in ROC analysis of baseline FGF-23 increased per tertile (Group 1 (creatinine ≤100 μmol/L): AUC 0.515; Group 2 (creatinine 101 to 142 μmol/L): AUC 0.675; Group 3 (creatinine ≥142 μmol/L): AUC 0.750).

**Table 3:** **Fibroblast growth factor 23 quartiles and hazard ratios with 95% confidence interval for long-term mortality adjusted for age, ejection fraction and baseline serum lactate**

	**Quartile 1**	**Quartile 2**	**Quartile 3**	**Quartile 4**
Overall cohort	1	1.02	1.59	2.34
		(0.44–2.37)	(0.78–3.24)	(1.12–4.88)
Creatinine ≥117 μmol/L	1	1.11	2.78	3.56
		(0.17–7.06)	(0.61–12.62)	(0.81–15.72)
Creatinine <117 μmol/L	1	0.89	0.95	1.42
		(0.33–2.38)	(0.36–2.49)	(0.30–6.82)

## Discussion

The present study aimed to validate and extend preliminary findings from a prior study on plasma levels of the phosphaturic hormone FGF-23 in CS: we first confirm profoundly increased FGF-23 plasma levels in patients with infarction-related CS in a larger, better-characterized patient cohort within the randomized IABP-SHOCK II trial [9]. Second, we were able to show that increased FGF-23 levels are associated not only with increased short-time (30-day) - as reported in our earlier study [9] - but also long-term (1-year) mortality. Third, the negative prognostic association of FGF-23 with both short- and long-time mortality persisted in multivariable analyses adjusted for several well-known prognostic factors. Fourth, the prognostic role of FGF-23 appears only to be relevant in patients with impaired renal function, as assessed by the baseline serum creatinine concentrations.

The phosphaturic hormone FGF-23 is profoundly increased in patients with CKD [24] and associated with cardiovascular events and mortality [10,11]. Recently, understanding has emerged that this relationship is also present in apparently healthy individuals from the general population [14], in patients with prevalent cardiovascular disease [10] and in patients with stable chronic systolic heart failure [12,13].

As a pathophysiological explanation for these epidemiological findings, Faul *et al*. could show that FGF-23 exerts direct harmful pro-hypertrophic effects on myocardial tissue *in vivo* and *in vitro* [16]. Taken together, there is evidence from clinical and experimental studies that FGF-23 may not only be a bystander, but a direct or indirect mediator of cardiovascular disease. However, a direct contribution of FGF-23 to myocardial disease is still under debate, after two further experimental studies failed to confirm a role of FGF-23 in left ventricular hypertrophy [20,25].

The present data provide further evidence to the proposed reverse causality between elevated FGF-23 and cardiovascular disease according to which high FGF-23 secretion (hyperphosphatonism) not (only) induces cardiovascular disease, but instead prevalent subtle or overt cardiovascular disease may induce FGF-23 secretion. This hypothesis is underscored by our present observations that - as in our previous study [9] - FGF-23 levels in CS patients exceeded by far those found in patients with stable CAD, or in individuals with mild-to moderate CKD. Thus, it is unlikely that patients recruited into the present study had a substantial elevation of FGF-23 before they suffered CS, even though we cannot present formal evidence for this assumption.

Interestingly, in several prospective epidemiologic studies, baseline FGF-23 levels did not predict future AMI [12,26] and in our previous study, patients with AMI without CS are characterized by FGF-23 levels comparable to those of stable CAD patients [9]. Thus, the profound rise in FGF-23 appears to be due to the neuroendocrinologic and/or hemodynamic impacts of CS rather than to the acute myocardial ischemia *per se*. A possible explanation for the increase in FGF-23 in CS might be the interplay between FGF-23, the RAAS [8,27] and the sympathetic activation [28]. Patients with CS are characterized by an activated RAAS and sympathetic system [29]. The increased RAAS activity downregulates Klotho, an important cofactor of FGF-23 signaling, and leads to an upregulation of FGF-23 [8]. Similarly, a role of sympathetic activation in FGF-23 secretion was proposed [28].

In the present study, the negative prognostic association of FGF-23 was not only observed with regard to short-term (30 days) but also to 1-year mortality. Furthermore, FGF-23 remained an independent predictor for mortality in a multivariate analysis including several factors known to affect risk of CS patients. Notably, regarding risk prediction of short-term mortality, FGF-23 was equally potent as serum lactate levels and combination of these two factors increased the diagnostic accuracy significantly.

Interestingly, the negative prognostic association of FGF-23 was only observed in the patients with serum creatinine concentrations at baseline above the median, whereas in patients below the median, FGF-23 levels did not differ between survivors and nonsurvivors. These results are in line with previously published epidemiologic data, where associations to left ventricular hypertrophy and outcome have generally been stronger among individuals with impaired renal function [18,30]. As one example, cross-sectional data from the Heart and Soul Study, which recruited 887 patients with stable CAD and history of AMI, found an association between FGF-23 and left ventricular hypertrophy in individuals with estimated glomerular filtration rate <60 ml/min/1.73 m^2^, but not in participants with intact renal function [20]. Patients with CKD are characterized by an activated vitamin D deficiency due to reduced 1α-hydroxylase activity. Low vitamin D has been shown to be a predictor of mortality in critically ill patients [31,32]. As vitamin D inhibits renin release, vitamin D deficiency is believed to increase RAAS activity. This, in turn, might further promote the previously mentioned negative interplay between the RAAS and FGF-23 in CS. Increased RAAS activity leads to increased FGF-23 levels, which, in turn further impairs vitamin D activation [8].

As a limitation to all epidemiologic studies, we cannot prove that elevated FGF-23 is causally linked to adverse outcome in CS. One potential mechanism might be the above-mentioned interplay between FGF-23 and vitamin D. Moreover, recent evidence suggests that FGF-23 may contribute to volume retention, which further aggravates CS [33]. Alternatively, high FGF-23 may reflect preexisting CKD, or more severe left ventricular disease, both of which directly contribute to poor outcome in CS. A further limitation of the study is its design as a single-center subanalysis of a multicenter study.

## Conclusions

In conclusion, our findings confirm in a large cohort study that patients with CS are characterized by profoundly increased FGF-23 levels. Beyond this, we show that in CS patients increased FGF-23 has a strong and independent negative prognostic association with short- and long-term outcome and that this association is observed only in patients with worse renal function. These results underline the need for future studies in order to clarify whether this association is causal or whether FGF-23 rather represents an innocent bystander, partly reflecting the underlying kidney damage, or more severe left ventricular disease.

## Key messages

- FGF-23 levels are an independent predictor of short- and long-term outcome in CS complicating AMI.
- These findings were only observed in patients with reduced kidney function.

## Abbreviations

AMI: acute myocardial infarction
AUC: area under the curve
CAD: coronary artery disease
CI: confidence interval
CKD: chronic kidney disease
CS: cardiogenic shock
EF: ejection fraction
FGF-23: fibroblast growth factor 23
HR: hazard ratio
IABP: intraaortic balloon pump
IQR: interquartile range
OR: odds ratio
PCI: percutaneous coronary intervention
RAAS: renin-angiotensin-aldosterone system
